# Pulsatile Myofilament Activity in Myotrem Myopathy Associated with Myogenic Tremor

**DOI:** 10.3390/ijms26115252

**Published:** 2025-05-30

**Authors:** Jennifer Megan Mariano, Laurin M. Hanft, Suhan Cho, Christopher W. Ward, Kerry S. McDonald, Aikaterini Kontrogianni-Konstantopoulos

**Affiliations:** 1 Department of Biochemistry and Molecular Biology, University of Maryland School of Medicine, Baltimore, MD 21201, USA; jennifer.mariano@som.umaryland.edu (J.M.M.); suhan.cho@som.umaryland.edu (S.C.); 2 Department of Medical Pharmacology and Physiology, University of Missouri School of Medicine, Columbia, MO 65212, USA; hanftl@health.missouri.edu; 3 Department of Orthopaedics, University of Maryland School of Medicine, Baltimore, MD 21201, USA; ward@som.umaryland.edu

**Keywords:** myogenic tremor, myosin binding protein-C slow, Myotrem, spontaneous oscillatory contraction, stretch activation

## Abstract

Myosin-binding protein C (MyBP-C) comprises a family of myofilament proteins that maintain sarcomeric structure and regulate actomyosin crossbridge cycling. Pathogenic variants in *MYBPC1*, the gene encoding the slow skeletal isoform (sMyBP-C), lead to a dominant congenital myopathy, termed Myotrem, characterized by muscle weakness, hypotonia, and a distinctive tremor of myogenic origin, in the absence of neuropathy. However, the molecular mechanism(s) of myogenic tremorgenesis is largely unknown. One potential mechanism is aberrant myofilament stretch activation, which is defined as a delayed increase in force after a rapid stretch. We utilized the Myotrem murine model harboring the pathogenic *MYBPC1* E248K variant to test the hypothesis that stretch activation is augmented in permeabilized Myotrem E248K soleus fibers. We found that stretch activation was significantly increased in E248K soleus muscle fibers. Interestingly, once submaximally Ca^2+^ activated, a subpopulation of slow-twitch E248K fibers exhibited spontaneous pulsatile sarcomere oscillations. This pulsing behavior generated a sinusoidal waveform pattern in sarcomere length, which often persisted on a timescale of minutes. These results align with sMyBP-C as key regulator of the synchronous activation of myofilaments by dampening both spontaneous oscillatory activity and stretch-dependent activation. We propose that the presence of sMyBP-C-E248K disrupts this regulation, thereby driving pathogenic myogenic tremors.

## 1. Introduction

Pathogenic variants in *MYBPC1*, the gene encoding the slow skeletal isoform of Myosin Binding Protein-C (sMyBP-C), are linked to a newly recognized form of congenital myopathy termed Myotrem (OMIM #618524). Myotrem is characterized by congenital hypotonia, early onset muscle weakness, skeletal deformities, and, most characteristically, tremor of myogenic origin [1]. The myogenic tremor is largely postural, accentuated with action, and is present mainly in the upper and lower extremities as well as the tongue and facial muscles [1]. sMyBP-C is ubiquitously expressed in skeletal muscle in both slow- and fast-twitch fibers, although in different amounts and isoform combinations [2]. Localized to the C-zone of the sarcomeric A-band [3], sMyBP-C is constitutively bound to the thick filament at its COOH-terminal end [4], while its NH_2_-terminus interacts dynamically with both myosin [5] and actin [6,7]. sMyBP-C plays a role in the assembly and maintenance of sarcomeric organization [8] and critically regulates actomyosin crossbridge cycling. Given that sMyBP-C expression is restricted to skeletal muscle and that Myotrem patients exhibit no neuropathy, the tremor is believed to originate in the muscle itself starting at the level of the sarcomere [1]. However, the molecular mechanisms of myogenic tremorgenesis remain unknown.

Stretch activation is a property of striated muscle whereby rapid mechanical stretch of an isolated muscle fiber leads to a delayed increase in force [9,10]. This stretch-activated response occurs in three phases. In phase 1, mechanical stretch strains actomyosin cross-bridges, generating a rapid increase in tension. In phase 2, tension rapidly decreases as strained cross-bridges detach. Finally, in phase 3, there is a delayed increase in muscle tension, known as stretch activation.

Stretch activation is often studied in insect flight muscles, where it is more pronounced and facilitates asynchronous muscle contraction with membrane depolarization [11]. However, stretch activation is also a property of vertebrae striated muscles and contributes to physiological function [9,12,13,14,15]. In cardiac myocytes, which rhythmically contract with each heartbeat, stretch activation is thought to contribute to length-dependent activation and the Frank–Starling mechanism [16]. Although the mechanisms of stretch activation are not fully understood, alterations in the levels or phosphorylation status of MyBP-C have been shown to regulate stretch activation in cardiac myocytes [13] and skeletal muscle fibers [15]. It seems plausible that aberrant stretch activation may be a potential mechanism contributing to myogenic tremor. Thus, we tested the hypothesis that stretch activation is augmented in permeabilized soleus muscle fibers from *MYBPC1* E248K knock-in (KI) Myotrem mice.

## 2. Results

### 2.1. Contractile Properties of Soleus Muscle Fibers

Permeabilized fibers from wild type (WT) and *MYBPC1* E248K knock-in (KI) soleus muscles were first evaluated for their morphometric characteristics and mechanical performance in relaxing and maximal Ca^2+^ activating solutions (Table 1). Fiber preparation length, width, resting sarcomere length, and passive tension were comparable between groups. However, maximal Ca^2+^ activated force was significantly reduced in E248K KI soleus fibers (Table 1), an observation consistent with similar measurements in permeabilized E248K KI extensor digitorum longus (EDL) fibers [17]. Moreover, the rate constant of force development after a slack–re-stretch maneuver was comparable between WT (pCa 4.5 *k_tr_* = 10.5 ± 1.1 s^−1^) and E248K KI (pCa 4.5 *k_tr_* = 10.3 ± 1.1 s^−1^) soleus fibers during maximal Ca^2+^ activation.

### 2.2. Stretch Activation Properties of Soleus Muscle Fibers

**Figure 1:**
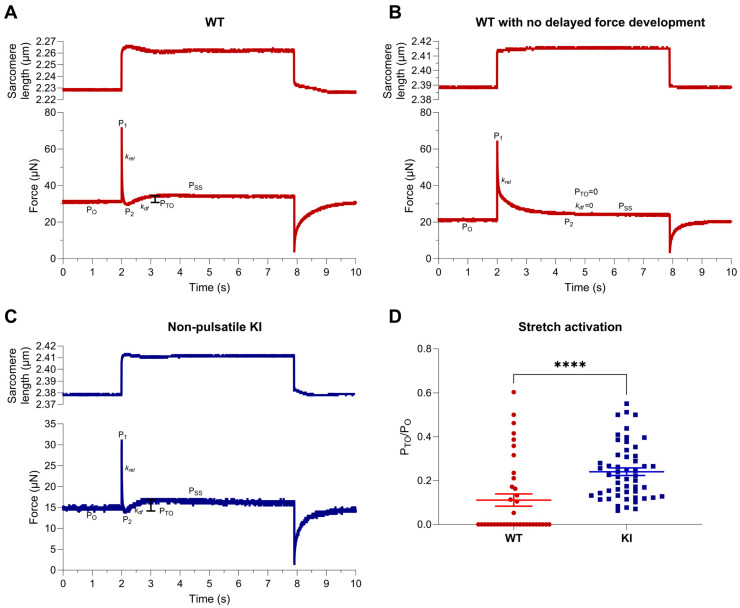
Stretch activation responses of permeabilized WT and E248K KI soleus fibers. (**A**–**C**). Representative sarcomere length and force traces during a stretch activation maneuver of a 1% sarcomere length stretch of permeabilized WT (**A**,**B**) and E248K KI (**C**) soleus fibers. Stretch activation is quantified as the delayed transient force overshoot (P_TO_) after sarcomere length stretch. On each trace, properties of the stretch-activated response are indicated, which include pre-stretch tension (P_o_), an initial force spike that coincides with stretch (P_1_), minimum of quick force decay (P_2_), rapid force decay rate (*k_rel_*), delayed force re-development rate (*k_df_*), and delayed force overshoot or stretch activation (P_TO_), before resolving to a new stretch-activated steady state tension (P_SS_). Fibers that did not exhibit delayed force redevelopment (**B**) had P_TO_ and *k_df_* values of zero. (**D**) Stretch activation was significantly increased in E248K KI soleus muscle fibers, as indexed by delayed force overshoot after stretch (P_TO_/P_O_). Values are calculated from *n* = 38 stretch maneuvers of *n*′ = 8 fibers from *n*″ = 5 WT mice and *n* = 53 stretch maneuvers of *n*′ = 11 fibers from *n*″ = 5 E248K KI mice. Data are expressed as mean ± SEM and statistical significance was determined by Mann–Whitney test; WT P_TO_/P_o_ = 0.111 ± 0.028; E248K KI P_TO_/P_o_ = 0.240 ± 0.017, **** *p* < 0.0001.

We next investigated whether stretch activation properties were augmented in E248K KI soleus fibers. Fibers were subjected to rapid step–stretches that yielded a 1–4% change in sarcomere length during submaximal Ca^2+^ activations, and the resulting triphasic stretch-activated response was recorded (Figure 1A–C). Stretches that failed to induce delayed force development (Figure 1B) were assigned a P_to_ value of zero and were included in the analysis. Stretch activation, quantified by P_to_/P_o_, was significantly greater in E248K KI soleus fibers compared to WT (WT P_to_/P_o_ = 0.111 ± 0.028; E248K KI P_to_/P_o_ = 0.240 ± 0.017, **** *p* < 0.0001; Figure 1D). Interestingly, all E248K KI fibers displayed stretch activation (i.e., delayed force transient after rapid stretch), whereas only ~50% of WT soleus fibers exhibited stretch activation, which was a major contributor to group differences.

**Figure 2:**
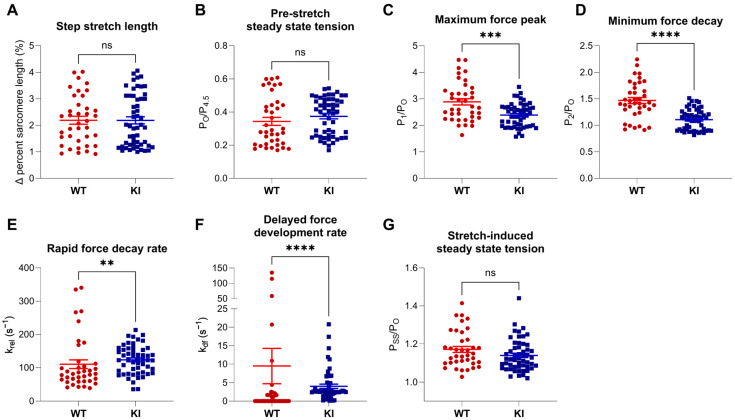
Stretch activation properties of permeabilized WT and E248K KI soleus fibers. Stretch activation properties of soleus fibers undergoing stretch maneuvers during submaximal Ca^2+^ activation (pCa 6.2–6.5). (**A**) Fibers underwent similar step stretches ranging from 1–4% sarcomere length (*p* = 0.94). (**B**) Pre-stretch steady state tension was similar between WT and E248K KI fibers, (*p* = 0.17). During the stretch maneuver, the calculated maximum force peak (**C**; *** *p* = 0.0004), minimum tension of force decay (**D**; **** *p* < 0.0001), rapid force decay rate (**E**; ** *p* = 0.0046), and delayed forced development rates (**F**; **** *p* < 0.0001) were all depressed in E248K KI fibers compared to WT. After the stretch, the resolved stretched-induced steady state tension remained similar between WT and E248K KI fibers (**G**; *p* = 0.10). Values are calculated from *n* = 38 stretch maneuvers of *n*′ = 8 fibers from *n*″ = 5 WT mice and *n* = 53 stretch maneuvers of *n*′ = 11 fibers from *n*″ = 8 KI mice. Data are expressed as mean ± SEM and statistical significance was determined by Welch’s *t*-test (**C**), or Mann–Whitney test if normality failed (**A**,**B**,**D**,**E**); ns: not significant.

Further evaluation of stretch activation parameters indicated that step–stretch length (Figure 2A) and pre-stretch steady state tension (Figure 2B) were similar between WT and E248K KI fibers. However, post-stretch, E248K KI fibers exhibited significantly reduced maximum force peak (P_1_/P_O_; Figure 2C) and minimum force decay (P_2_/P_O_; Figure 2D) tension, faster rapid force decay (*k_rel_*, Figure 2E) and slower delayed force development (*k_df_*, Figure 2F) kinetics, while stretch-induced steady state tension (P_SS_/P_O_, Figure 2G) remained comparable to WT. Taken together, the observed differences in stretch activation parameters implicate altered interactions between mutant sMyBP-C and the sarcomeric thick and thin filaments. For instance, the difference between P_2_ and P_TO_ is primarily a function of the rate of phase 2 decay (i.e., rapid force decay, *k_rel_*) compared to the rate of phase 3 increase (i.e., delayed force development, *k_df_*). Thus, the significantly reduced P_2_/P_O_ values in the E248K KI fibers in phase 2 indicate faster detachment of strained cross-bridges in the presence of similar or slightly lower rates of cross-bridge force development (*k_df_*) in phase 3. Moreover, the lower P_1_/P_O_ values in the E248K KI fibers are also consistent with faster cross-bridge attachment rates but may also arise from reduced stiffness per bridge.

### 2.3. Pulsatile Activity of a Subpopulation of E248K KI Soleus Muscle Fibers

**Figure 3:**
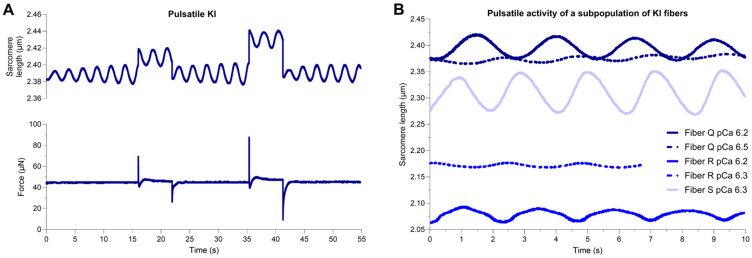
Pulsatile SPOC-like activity in permeabilized E248K KI soleus fibers. (**A**,**B**) A sub-population of E248K KI permeabilized soleus fibers exhibited pulsatile sarcomere length oscillations during submaximal Ca^2+^ activation. The pulses oscillated rhythmically at ~0.5 Hz and persisted throughout the stretch activation protocol. Pulsatile fibers were not observed in the WT group under any tested condition. (**A**) Representative sarcomere length and force traces of a pulsatile E248K KI fiber undergoing two stretch maneuvers at pCa 6.2. Data are shown over a 55 s period with a sampling rate of 250 Hz and 1000 Hz for sarcomere length and force, respectively. (**B**) Sarcomere length traces of E248K KI fibers that exhibit unsolicited pulsatile activity in submaximal Ca^2+^ solutions (pCa 6.2–6.5). Navy, periwinkle, and bright blue represent traces from different (*n* = 3) pulsatile soleus fibers from three E248K KI mice. Data shown are over a 10 s period of steady-state equilibrium with a sampling rate of 250 Hz.

Permeabilized fibers from WT and E248K KI soleus muscles were subsequently subjected to mechanical measurements in submaximal Ca^2+^ conditions. All eight WT fibers and eight of 11 E248K KI fibers remained at constant sarcomere length during sub-maximal Ca^2+^ activation, consistent with prototypic behavior (Figure 1A–C and Supplemental Video S1). However, a subpopulation, three of 11, of E248K KI fibers exhibited pulsatile spontaneous oscillatory contraction (SPOC)-like behavior during sub-maximal Ca^2+^ activation (Figure 3 and Supplemental Videos S2–S4). This unsolicited pulsatile SPOC-like activity consisted of rhythmic oscillations in sarcomere length that lasted for minutes and persisted during subsequent small rapid mechanical stretches (Figure 3A). Sarcomere length traces of these pulsatile E248K KI fibers revealed a unique sinusoidal pattern with temporally equivalent lengthening and shortening phases, reaching a consistent amplitude with each period. Despite this internal consistency, amplitude and frequency values varied amongst the three pulsatile E248K KI fibers (Figure 3B). Pulse amplitude and frequency were also altered within the same fiber in a pCa-dependent manner, where lower Ca^2+^ concentrations reduced pulse amplitude and frequency (Figure 3B). Pulsatile behavior was not observed in pCa 9.0 relaxing solution, where sarcomere lengths of relaxed fibers remained constant.

### 2.4. Fiber-Type Specific Stretch Activation Properties

**Figure 4:**
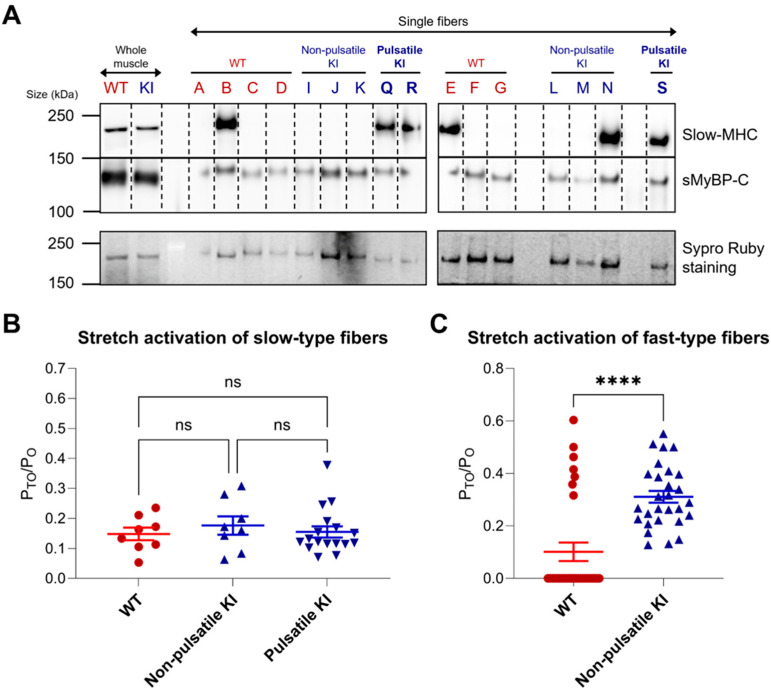
Fiber typing and stretch activation properties of WT and E248K KI soleus fibers. (**A**) Single fiber SDS-gel electrophoresis and Western blotting were performed on WT, non-pulsatile E248K KI, and pulsatile E248K KI fibers after mechanical measurements. Lysates from whole soleus muscles from WT and E2348K KI mice were included as positive controls, and SYPRO Ruby was used to visualize total protein levels. Blots were probed for slow myosin heavy chain (MHC) and sMyBP-C. Slow-MHC was positively identified in 2/7 (29%) WT, 1/6 (17%) non-pulsatile E248K KI, and 3/3 (100%) pulsatile E248K KI fibers by Western blotting. (**B**,**C**) Post-hoc analysis of stretch activation properties were stratified according to fiber type and pulsatile activity. Fiber type was defined by mechanical properties with slow fibers exhibiting a *k_tr_* value less than 7 s^−1^ and fast fibers displaying a *k_tr_* values greater than or equal to 7 s^−1^. (**B**) Stretch activation (P_TO_/P_O_) was comparable among WT, non-pulsatile E248K KI, and pulsatile E248K KI type-I slow fibers. Data are expressed as mean ± SEM. Values are calculated from *n* = 8 stretch maneuvers of *n*′ = 2 WT & *n*′ = 2 non-pulsatile E248K KI fibers, and *n* = 17 stretch maneuvers of *n*’ = 3 pulsatile E248K KI type-I slow fibers. Statistical significance was determined by Kruskal–Wallis one-way ANOVA; ns: not significant. (**C**) While none of the E248K KI fast type-II fibers exhibited pulsatile activity, they developed enhanced stretch activation when compared to their WT counterparts. Values are calculated from *n* = 30 stretch maneuvers of *n*′ = 6 WT and *n* = 28 stretch maneuvers of *n*′ = 6 E248K KI fast type-II fibers. Statistical significance was determined by Mann–Whitney test; **** *p* < 0.0001.

Given the spontaneous pulsatile activity observed in a subset of E248K KI soleus fibers, we aimed to explore fiber characteristics that potentially contribute to pulsatile behavior. Soleus fibers underwent immunoblot analysis to determine fiber-type by probing for the slow isoform of myosin heavy chain (MHC) and sMyBP-C, while SYPRO Ruby stain was used to evaluate total protein levels in the tested fibers (Figure 4A). Interestingly, all three pulsatile E248K KI fibers were identified as slow type-I based on their positive expression of slow-MHC, a finding corroborated by our mechanical measurements, where all pulsatile E248K KI fibers exhibited a slower tension re-development rate constant (*k_tr_*) in maximal Ca^2+^ activating conditions of less than 7 s^−1^ (Table 1). Notably, one of six non-pulsatile E248K KI fibers was also type-I, suggesting that while a slow fiber type-I background may be necessary for E248K to elicit pulsatile behavior, it is not solely sufficient.

Considering the enhanced stretch activation observed in the E248K KI soleus fibers compared to WT (Figure 1D), we proceeded to assess the stretch activation characteristics relative to fiber type. Fiber type was classified based on mechanical properties, with a *k_tr_* value less than 7 s^−1^ indicating slow fibers, and a *k_tr_* value greater than or equal to 7 s^−1^ indicating fast fibers. No difference in stretch activation (P_to_/P_o_) was observed among WT, non-pulsatile E248K KI, and pulsatile E248K KI slow type-I fibers (Figure 4B). In contrast, E248K KI fast type-II fibers exhibited significantly enhanced stretch activation compared to their WT counterparts (Figure 4C). Interestingly, all WT fibers that lacked delayed force development were of the fast type. Thus, although stretch activation does not appear to contribute to the pulsatile activity of slow type-I E248K KI fibers per se, the elevated stretch activation observed in fast type-II E248K KI fibers may augment the muscle’s overall response to stretch, potentially contributing to the propagation of the pulsatile behavior present in a subgroup of slow type-I Myotrem fibers.

## 3. Discussion

Dominant missense variants of *MYBPC1*, the gene encoding sMyBP-C, are linked to a new myopathy termed Myotrem (OMIM #618524), associated with tremor [1]. Given the absence of neuropathy in individuals with Myotrem [1], and the restricted expression of *MYBPC1* in skeletal muscle, this characteristic tremor is believed to originate within the muscle itself [1,17]. Despite this understanding, the mechanism responsible for myogenic tremor has remained unclear. To study Myotrem, we generated a KI mouse model carrying the dominant *MYBPC1* E248K variant [17]. Consistent with the pathological manifestations of Myotrem seen in humans, heterozygous E248K KI mice present with skeletal muscle weakness, manifesting as reduced force production and suppressed contractility kinetics, as well as early-onset tremor [17]. Considering that neuromuscular diseases often display sex-dimorphic presentation [18], our studies exclusively focused on males. Nonetheless, since *MYBPC1* E248K Myotrem mice exhibit no evidence of sex dimorphism at young ages [17], similar results might be expected of female mice as well.

**Figure 5:**
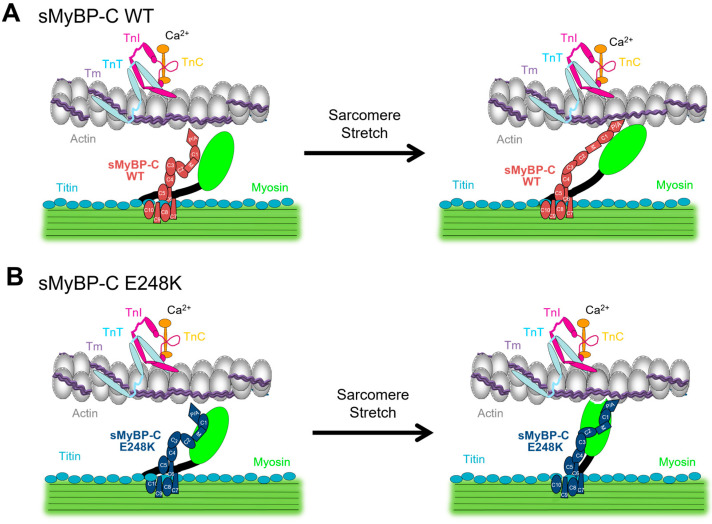
Proposed model of enhanced stretch activation in the presence of the *MYBPC1* E248K Myotrem variant. (**A**) In submaximal Ca^2+^ conditions, stretch may allow for lateral displacement of thick and thin filaments, promoting crossbridge formation as part of the stretch activation response, which is (at least partially) regulated by sMyBP-C. (**B**) Introduction of the *MYBPC1* E248K variant, enhances binding of sMyBP-C to myosin. This potentially allows sMyBP-C to provide structural support, thereby upon stretch, myosin is more likely to engage in crossbridge formation leading to enhanced stretch activation.

We first postulated that augmented stretch activation, which is defined as a delayed increase in force after a rapid stretch, may contribute to myogenic tremorgenesis. We therefore tested the hypothesis that stretch activation is increased in permeabilized E248K KI soleus muscle fibers. We report enhanced stretch activation in fast type-II E248K KI soleus muscle fibers when compared to their WT counterparts. Although the exact mechanism(s) of stretch activation remains unclear, alterations in the expression or phosphorylation profile of MyBP-C influence stretch activation in both cardiac and skeletal muscles [13,15] (Figure 5A). Our prior work has shown that the E248K variant significantly increases myosin binding [1]. We therefore postulate that upon stretch, this enhanced myosin binding may provide structural support, facilitating myosin displacement towards actin, thereby promoting crossbridge recruitment via mechanotransduction (Figure 5B).

Unexpectedly, we also identified a subset of slow type-I E248K KI fibers that exhibit rhythmic, pulsatile sarcomere oscillations during submaximal Ca^2+^ activation. We postulate that these pulsatile slow type-I E248K KI fibers may serve as initiators of the tremor, with the autonomous oscillatory signal being propagated and amplified via enhanced stretch activation in neighboring fast type-II fibers, ultimately producing a recordable tremor. The intrinsic pulsatile SPOC-like phenotype we uncovered in a subpopulation of E248K KI slow type-I fibers is independent of neurological input or stimulation through canonical excitation–contraction coupling. Notably, SPOC is typically characterized by a saw-tooth pattern of rapid lengthening and slow shortening cycles [12], which contrasts with the highly regular sinusoidal pattern of contraction and persistence exhibited by pulsing E248K KI slow type-I fibers. A case of highly synchronous SPOC-like activity has been reported in permeabilized rabbit psoas fibers under isotonic conditions [19], which mirrors the regularity of the pulsatile activity observed in E248K KI slow type-I fibers. However, this synchronous SPOC-like activity persisted on a timescale of seconds, whereas the pulsatile behavior we describe in E248K KI slow type-I fibers spans a timescale of minutes.

Pulsatile activity in E248K KI slow type-I fibers was dormant in relaxing solution, present in submaximal Ca^2+^ solutions, and typically absent at maximal Ca^2+^ concentration. Consequently, Ca^2+^ is required for pulsing, but pulsatile behavior is mostly distinct from maximal Ca^2+^ activated contraction. This finding aligns with Myotrem tremor on a physiological level, as tremor is mainly absent at rest but induced by posture, action and intention [1,20], which largely occur at submaximal activation [21]. Pulse frequency and amplitude also varied within pulsatile fibers in a pCa-dependent manner, as well as among pulsatile fibers. This supports the hypothesis of multiple tremor initiators within the body’s musculature, consistent with dual-channel electromyography (EMG) recordings in Myotrem patients revealing temporally asynchronous tremors between the two legs and tremor of incongruent frequency between the two arms [1].

Recent work has postulated that cardiac MyBP-C may regulate SPOC generation, as removal of its NH_2_-terminal region in cardiac myocytes leads to an increase in SPOC activity [22], prompting a newly proposed role of MyBP-C as a SPOC “wave breaker” [12]. Harris argued that removal of the NH_2_-terminus of cardiac MyBP-C, the regulatory center of the protein, accentuates SPOC generation by accelerating the detachment of strained cross-bridges [12,22], an idea consistent with our findings in E248K KI soleus fibers, which demonstrate reduced minimum force decay (P_2_/P_O_) during stretch activation. Relatedly, the expression levels and phosphorylation profile of the cardiac and slow skeletal MyBP-C proteins were found to modulate stretch activation responses in permeabilized cardiac and skeletal muscle fibers [13,15,23]. Thus, it is conceivable that pathogenic variants in *MYBPC1* may give rise to a SPOC-like phenotype.

Importantly, while all pulsatile E248K KI fibers are slow type-I, not all slow type-I E248K KI fibers display SPOC-like activity, indicating that a slow type-I fiber is likely necessary but not sufficient to elicit pulsatile activity. Moreover, while stretch activation among WT, E248K KI non-pulsatile, and E248K KI pulsatile slow type-I fibers is comparable, E248K KI fast type-II fibers display significantly enhanced stretch activation relative to their WT counterparts, with only ~50% of WT fast type-II fibers (versus ~100% of E248K KI fast type-II fibers) demonstrating the capacity to generate such a response. We therefore posit that pulsatile and non-pulsatile E248K KI fibers may serve distinct roles in myogenic tremorgenesis, yet likely work in concert to predispose dyssynchronous muscle fiber activity. When partially activated in submaximal Ca^2+^ conditions induced by posture or intention, a subpopulation of slow type-I Myotrem fibers exhibits spontaneous pulsatile activity. This mechanical perturbation may be transmitted to adjacent fast type-II fibers displaying enhanced stretch activation. As these fast type-II fibers develop force and contract in response to stretch, they may propagate the pulsatile activity initiated by the slow type-I fibers, resulting in the generation of recordable tremor.

While this is an intriguing notion, there are several unresolved questions, including the identification of the intrinsic characteristics of the potentially distinct fiber subsets. The molecular complexity of the sMyBP-C subfamily, encompassing >14 splice variants in humans expressed in varying combinations, ratios and amounts across individual muscles, fibers, and likely sarcomeres [2], that may undergo phosphorylation at both constitutive and variant-specific sites [24], likely play a key role in defining the disparate fiber subpopulations within a muscle. Moreover, the EMG patient studies describe preservation in tremor frequency upon loading, suggesting some degree of central nervous system (CNS) involvement in the observable tremor [1]. The muscle spindle and Golgi tendon organ serve as sensory receptors in skeletal muscle, which, upon stretch or compaction due to force generation, send an afferent signal to the CNS [25,26]. Thus, it is plausible that in pathological conditions, such as Myotrem, pulsatile activity of a fiber subset coupled with enhanced stretch activation of adjacent fibers may affect proprioception, resulting in propagation of a measurable tremor on the organ level via sensory receptors. Further studies ranging from quantitative modeling of myofilament regulation to measurements of contractile activity within a single fiber and contractile behavior between adjacent muscle fibers, will be necessary to fully decipher the etiologies of myogenic tremorgenesis.

Currently, there are no effective treatments for patients diagnosed with Myotrem. β-blockers, commonly prescribed to treat heart rhythm disturbances and essential tremor, and primidone, typically used to control epileptic seizures, have proven ineffective in treating Myotrem tremor. Further, other treatments used to mitigate neurogenic tremor, such as Levodopa for Parkinson’s disease [27], are unlikely to alleviate myogenic tremor, which occurs in the absence of neuropathy. Our studies highlight a potential mechanism underlying myogenic tremor, offering valuable insights for designing targeted therapies for myogenic tremorgenesis.

## 4. Materials and Methods

### 4.1. Study Approval

All animal work, including housing, husbandry, animal care, and monitoring, were conducted under protocols approved by the Institutional Animal Care and Use Committee of the University of Maryland School of Medicine. The generation of the Myotrem C57BL/6J murine model expressing the murine *Mybpc1* E249K substitution corresponding to the human E248K variant was previously described [17]. KI Myotrem mice are referred to as the *MYBPC1* E248K KI murine model to avoid confusion. Studies use healthy two-month-old WT and heterozygous E248K KI male mice that have not undergone any previous procedures. To maximize consistency, this study focuses on measurements from male mice, as many neuromuscular disorders are known to affect males and females differently [18]. Sample sizes were determined from power analysis and information from previous studies [15].

### 4.2. Solutions

Relaxing solution consisted of 1 mM dithiothreitol (DTT), 100 mM KCl, 10 mM imidazole, 2.0 mM EGTA, 4.0 mM ATP, and 1 mM free (5 mM total) MgCl_2_, supplemented with Pefabloc protease inhibitor (Supelco, MilliporeSigma, Burlington, MA, USA). Basal Ca^2+^ activating solution contained 7.00 mM EGTA, 20 mM imidazole, and 14.50 mM PCr. Minimal Ca^2+^ activating solution (pCa 9.0) was supplemented with 5.42 mM MgCl_2_, 72.37 mM KCl, 0.016 mM CaCl_2_, and 4.7 mM ATP, ionic strength 180 mM. Maximal Ca^2+^ activating solution (pCa 4.5) was supplemented with 5.26 mM MgCl_2_, 60.25 mM KCl, 7.01 mM CaCl_2_, and 4.81 mM ATP, ionic strength 180 mM. Submaximal activating solutions of intermediate pCa values ranging between 5.9 and 6.5 were made by combining the appropriate ratios of minimal and maximal Ca^2+^ solutions.

### 4.3. Fiber Preparation and Experimental Setup

Fiber preparation, experimental setup, stretch-activated force measurements, and data analysis occurred as described previously [15] by a blinded experimenter. In short, mice were euthanized via asphyxiation using carbon dioxide followed by cervical dislocation. Soleus muscle was isolated and secured at each tendon to a toothpick to preserve sarcomere length. The tissue was incubated in a 1:1 relaxing solution:glycerol at −20 °C to allow for chemical skinning as previously reported [15,17,28,29,30,31,32]. As in the aforementioned studies, no detergents were applied to these fibers bundles; this yields the possibility of internal organellar calcium stores [33]. On the day of experimentation, single fibers were dissected, and the ends of the fiber were secured to stainless steel troughs with 3-0 monofilament sutures and subsequently mounted to a capacitance gauge force transducer (model 403, sensitivity of 20 mV/mg, resonant frequency of 600 Hz; Aurora Scientific, St. Aurora, ON, Canada) and a length control fitted to a DC torque motor (model 308c; Aurora Scientific). The apparatus was mounted over an Olympus IX70 inverted microscope (with a ×40 objective (Olympus UWD 40, 0.55 N.A., Olympus, Center Valley, PA, USA) on a pneumatic vibration isolation table. Morphological measurements were made while the fiber preparation was relaxed. Sarcomere length was monitored continuously using the IonOptix SarcLen video system and fast Fourier transform analysis of a ~220 × 30 μm region of interest and all measurements were performed at 15 ± 1 °C. A total of eight fibers from five WT mice and 11 fibers from five KI mice were harvested for experimentation (Table 1).

### 4.4. Slack–Re-Stretch Protocol

Slack–re-stretch experiments were performed as described previously [15,34] to measure crossbridge kinetics in maximal and submaximal Ca^2+^ activating conditions (pCa 4.5). In brief, permeabilized fibers were placed in maximal Ca^2+^ activating solution (pCa 4.5) at optimal length and steady state was allowed to develop. Subsequently, the fiber was slackened, rapidly re-stretched to mechanically detach cross-bridges to a value slightly greater than optimal length for ~2 ms and returned to optimal muscle length allowing for crossbridge formation and force redevelopment. The resulting force curve was fit to a single exponential equation:

(1) $$F=F_{res}+F_{max}(1-e^{\left({-k}_{tr}\right)x})$$

where the force, *F*, at any time *x*, is related to the residual tension immediately after the slack–re-stretch maneuver (*F_res_*), the maximal isometric force (*F_max_*), and the rate constant of force development, (*k_tr_*). For each fiber, the slack–re-stretch maneuver was performed at the onset and conclusion of the mechanical protocols and the reported *k_tr_* value is the average of these two repeats.

### 4.5. Step–Stretch Protocol and Data Analysis

Following the initial slack–re-stretch protocol, the fiber was transferred to submaximal Ca^2+^ activating solution and subjected to a step–stretch protocol to investigate stretch activation properties. During each submaximal Ca^2+^ activation, the fiber was allowed to reach steady-state tension (P_O_) and then up to five separate, fixed voltage (0.05, 0.10, 0.20, 0.30, 0.40 V, motor calibration 235 µm/V) fiber length stretches were applied, which yielded sarcomere length stretches ranging from ~0.5 to 8% sarcomere length. For analysis, group data was pooled for Ca^2+^ activated forces between 20 and 60% maximal and sarcomere length stretches of 1 to 4% since we previously did not observe independent variable-dependence of stretch activation parameters over these ranges [15]. The rapid fiber stretches elicited changes in force that exhibited the prototypical triphasic stretch-activated response: immediate sharp increase in force (P_1_) in phase 1; subsequent decrease in force (P_2_) in phase 2, that occurs with a kinetic constant of k_rel_; and stretch activation in phase 3, which is the slow delayed development in force (P_TO_) following stretch that occurs at a time constant of k_df_. The force trace during this phase was fit to a single exponential rise to maximum equation:

(2) $$F=P_{2}+A(1-e^{({-k}_{df})x})$$

where *A* is the amplitude of the exponential phase and *F* is the force at *x* seconds of phase 3. The fiber then reached a new steady state tension. This stretch was held for about 6 sec before the fiber was shortened to its original sarcomere length (Figure 1A–C). The fiber was allowed to reach steady-state tension again at this original length before undergoing subsequent step stretches, and the stretch-activated response of each stretch was recorded. For each fiber, this protocol was repeated in two different submaximal Ca^2+^ solutions: one that elicited ~50% of the fiber’s maximal Ca^2+^ activated force (pCa where P_O_/P_pCa4_._5_ = 50%), and another that elicited ~25% of the fiber’s maximal Ca^2+^ activated force (pCa where P_O_/P_pCa4_._5_ = 25%). Stretch activation tension measurements, i.e., phase 1 maximum force (P_1_), phase 2 minimum force (P_2_), stretch-activated force development (P_TO_), and stretch-induced steady state force (P_SS_) were normalized to the initial steady state force (P_O_).

### 4.6. Single Fiber Immunoblot Analysis

Following mechanical measurements, single permeabilized fibers were incubated in relaxing solution with sodium dodecyl sulfate (SDS). Individual fibers were fractionated by SDS-PAGE using a 4–12% tris-acetate gel (Invitrogen, Waltham, MA, USA) and transferred to nitrocellulose membrane. Lysates from whole soleus WT and KI muscles were included as positive controls and total protein was visualized using SYPRO Ruby protein staining (Thermo Scientific, Waltham, MA, USA). The blot was subsequently cut into strips and probed with antibodies against the slow isoform of myosin heavy chain (mouse monoclonal, Sigma-Aldrich, M8421, 1:1000, Millipore Sigma, St. Louis, MO, USA) and sMyBP-C (rabbit polyclonal, Sigma-Aldrich, SAB3501005, 1:1000, Millipore Sigma, St. Louis, MO, USA) at 4 °C overnight. After washing, the strips were incubated with the appropriate horseradish peroxidase-conjugated secondary antibody (Cell Signaling Technology, Danvers, MA, USA), followed by ECL substrate (Thermo Scientific, Waltham, MA, USA). Poorly preserved fibers were excluded from analysis and thus reported immunoblot data reflects seven of eight WT fibers, six of eight quiescent KI fibers, and three of three pulsing KI fibers.

### 4.7. Statistical Analysis

Statistical tests, sample sizes (*n*), and *p*-values are provided in figure legends. Shapiro–Wilk’s test was used to assess normality, and an F test was used to compare variances. Comparisons between two normal datasets of similar variance were performed using Student’s 2-tailed *t*-test, whereas comparisons of datasets that failed the normality or variance tests were performed with Mann–Whitney test and Welch’s *t*-test, respectively. Comparisons between three non-normal datasets were performed using Kruskal–Wallis test. Statistical analysis was performed using SigmaPlot (version 14; Palo Alto, CA, USA).and GraphPad Prism (version 10.4.2; San Diego, CA, USA). Values are expressed as mean ± SEM; ns: not significant; * *p* < 0.05; ** *p* < 0.01, *** *p* < 0.001, and **** *p* < 0.0001.

## Figures and Tables

**Table 1:** Cellular Characteristics of WT vs *MYBPC1* E248K KI Permeabilized Soleus Skeletal Muscle Fibers.

		Fiber Length (µm)	Fiber Width_air_ (µm)	Sarcomere Length (µm)	Passive Tension (kN/m^2^)	Maximal Tension (kN/m^2^)	pCa 4.5 k_tr_ (s^−1^)
WT	*n*′ = 8	911 ±71	39 ± 2	2.36 ± 0.03	3.29 ± 0.49	93 ± 5	10.5 ± 1.1
KI	*n*′ = 11	939 ± 60	44 ± 1	2.32 ± 0.03	2.56 ± 0.32	64 ± 6 *	10.3 ± 1.1

Fiber characteristics were compared via Student’s *t*-test. Values are means ± SEM. * *p* < 0.05. *n*′: number of WT and E238K KI fibers.

## Data Availability

Source data, including raw data values and original blot images, and Supplemental Videos, can be accessed at DOI: 10.6084/m9.figshare.28801487.

## Supplementary Materials

The supporting information can be downloaded at: https://www.mdpi.com/article/10.3390/ijms26115252/s1.

## Abbreviations

The following abbreviations are used in this manuscript:

CNS	Central nervous system
EDL	Extensor digitorum longus
k_df_	Delayed force development rate
KI	Knock-In
k_rel_	Rapid force decay rate
MHC	Myosin Heavy Chain
MyBP-C	Myosin binding protein-C
P_O_	Pre-stretch steady state tension
P_SS_	Stretch-induced steady state tension
P_TO_	Stretch activation
P_1_	Maximum force peak
P_2_	Minimum force decay
sMyBP-C	Slow skeletal myosin binding protein-C
SPOC	Spontaneous oscillatory contraction
WT	Wild type
